# Respiratory muscle weakness in acute heart failure patients

**DOI:** 10.1186/cc10172

**Published:** 2011-06-22

**Authors:** LHR Gonçalves, P Veríssimo, K Timenetsky, T Figueiredo, A Yang, T Andre, M Nagano, C Alexandre, A Goedert, R Caserta, E Silva

**Affiliations:** 1 Hospital Israelita Albert Einstein, São Paulo - SP, Brazil

## Background

Respiratory muscle weakness has been arbitrarily defined as a maximum inspiratory pressure lower than 70% of the predictive value. Patients with chronic heart failure have 30 to 50% prevalence of respiratory muscle weakness, and so far there is no evidence of this prevalence in patients hospitalized with acute heart failure.

## Objective

To evaluate maximum inspiratory pressure and the prevalence of respiratory muscle weakness in hospitalized patients with acute heart failure.

## Methods

A cohort study, performed at Hospital Israelita Albert Einstein in acute heart failure patients admitted to our hospital. We excluded patients with chronic pulmonary disease, neurological and neuromuscular disorders, postoperative period and those that needed an orotracheal tube. Patients after respiratory and hemodynamic stability were submitted to a maximum inspiratory pressure (MIP) measurement by a manuvacuometer. Measurement was performed using a facial mask and unidirectional valve with the patient positioned at 45°. We also collected demographic data, brain natriuretic peptide hormone (BNP), ejection fraction estimated by echocardiogram and use of non-invasive ventilation. MIP was measured at two moments, the first measurement as soon as patients were clinically stable and the second measurement before hospital discharge.

## Results

We evaluated 50 patients, with a mean age of 75 years (95% CI = 72 to 78.8), mostly male patients (78%, 39 patients), mean ejection fraction of 0.33 (95% CI = 0.31 to 0.35), and 93.5% had ejection fraction lower than 0.45. At hospital admission, 24 patients used NIV (55.8%), and the BNP median value was 726.5 pg/ml (range of 217 to 2,283 pg/ml). The first MIP measurement showed a median of -52 cmH_2_O (range of -20 to -120 cmH_2_O), with 35 patients (70%) presenting MIP lower than 70% of the predictive value. Time to the first measurement had a median of 3.5 days (range of 1 to 22 days). At hospital discharge, the median MIP was -53 cmH_2_O (range of -20 to -150 cmH_2_O), and maintained 70% of patients with MIP lower than 70% of the predictive value. There was no significant difference between initial and hospital discharge MIP (*P *= 0.806). Median hospital length of stay was 11 days (range of 4 to 36 days).

## Conclusion

Hospitalized patients with acute heart failure have a high prevalence of respiratory muscle weakness, and maintain weakness even after clinical stabilization.

